# Northern Hemisphere Urban Heat Stress and Associated Labor Hour Hazard from ERA5 Reanalysis

**DOI:** 10.3390/ijerph19138163

**Published:** 2022-07-03

**Authors:** Shih-Yu Lee, Shih-Chun Candice Lung, Ping-Gin Chiu, Wen-Cheng Wang, I-Chun Tsai, Thung-Hong Lin

**Affiliations:** 1 Research Center for Environmental Changes, Academia Sinica, Taipei 11529, Taiwan; sclung@rcec.sinica.edu.tw (S.-C.C.L.); phdzen@gate.sinica.edu.tw (W.-C.W.); ictsai@rcec.sinica.edu.tw (I.-C.T.); 2 Geophysical Institute, University of Bergen, 5020 Bergen, Norway; tony.blc@gmail.com; 3 Institute of Sociology, Academia Sinica, Taipei 11529, Taiwan; zoo42@gate.sinica.edu.tw

**Keywords:** heat stress, wet-bulb globe temperature, urban population exposure, labor hour reduction

## Abstract

Increasing surface air temperature is a fundamental characteristic of a warming world. Rising temperatures have potential impacts on human health through heat stress. One heat stress metric is the wet-bulb globe temperature, which takes into consideration the effects of radiation, humidity, and wind speed. It also has broad health and environmental implications. This study presents wet-bulb globe temperatures calculated from the fifth-generation European Centre for Medium-Range Weather Forecasts atmospheric reanalysis and combines it with health guidelines to assess heat stress variability and the potential for reduction in labor hours over the past decade on both the continental and urban scale. Compared to 2010–2014, there was a general increase in heat stress during the period from 2015 to 2019 throughout the northern hemisphere, with the largest warming found in tropical regions, especially in the northern part of the Indian Peninsula. On the urban scale, our results suggest that heat stress might have led to a reduction in labor hours by up to ~20% in some Asian cities subject to work–rest regulations. Extremes in heat stress can be explained by changes in radiation and circulation. The resultant threat is highest in developing countries in tropical areas where workers often have limited legal protection and healthcare. The effect of heat stress exposure is therefore a collective challenge with environmental, economic, and social implications.

## 1. Introduction

Ground temperatures all over the world and the intensity of environmental heat stress have increased in the past few years due to climate change and, based on current climate model projections, will continue to rise in future [1]. Climate change will prompt increases in temperature extremes, in addition to overall warming. Heat stress resulting from extremely warm temperatures has a profound influence on human beings, wildlife, and agriculture [2]. It is estimated that extreme heat caused more than two thousand deaths in the United States from 2006 to 2010 [3], as well as hundreds of millions of dollars in annual losses to the cattle industry alone [4]. Global annual crop production has already been impacted by climate warming [5].

The exposure of the population, especially in urban settings, to this heat stress, which is on the order of hundreds of millions since 2000 [6], has great health, societal, and economic impacts [7,8,9]. Many studies have demonstrated the adverse influence of heat stress on the human body, causing dehydration, reduction in cardiovascular capacity, and heat exhaustion [10]. The risk is mainly caused by more metabolic heat generated inside the human body than can be dissipated to the ambient environment through evaporative cooling. Generation of body heat is proportional to the level of physical activity, while dissipation is a function of environmental factors such as temperature, humidity, and wind speed [11]. These factors have to be combined for an accurate quantification of the danger of heat stress. To this end, various metrics, such as the heat index, universal thermal climate index, discomfort index, and predicted heat strain have been developed. They have been shown to have a strong correlation with the wet-bulb globe temperature (WBGT) [12,13].

The WBGT has been widely used by military and occupational safety agencies as an indicator to assess exposure risk because of heat stress, not just mortality and morbidity outcome but also in relation to human injuries (e.g., see Reference [14]). The WBGT includes environmental-exposure-response factors and has been used to manage safe work and activity load in hot environments. The management of exposure to heat stress often involves a work/rest time cycle to mitigate heat-related illness, as recommended by the ACGIH. Increasing global temperatures and the intensification of heat stress directly lead to reductions in labor capacity and measures to maintain occupational safety [7]. Recent impact studies demonstrate heat-induced economic cost [9,15]. Based on daily temperature variability, an extra degree of elevation in temperature results in an average 5% reduction in the regional economic growth rate, up to a 12% reduction in low-latitude and low-income regions, thus making these regions particularly vulnerable to climatic warming.

Studies on the sub-continental scale, using reanalysis products, have been hampered by their low spatial model resolution. However, the proliferation of reanalysis products with high enough spatial resolution has finally made urban-scale research realistic. While conventional analyses focus on physical parameters such as changes in temperature, precipitation, and circulation, we take a new approach by using a comprehensive global model output that contains all necessary parameters to calculate the WBGT and quantify heat stress.

In the present study, we used ERA5 reanalysis to explore one of the consequences of exacerbated heat stress: potential labor-time reduction in major urban areas in the northern hemisphere in response to WBGT changes over a period of ten years. The period from 2010 to 2020 has been the hottest decade on instrumental record, and the changes in heat stress during that time will most likely predict possible decadal variability in the future warming climate.

## 2. Data and Methodology

The WBGT has conventionally been calculated from in situ instrumental measurements to represent heat stress conditions in a specific environment. In the past, most of the available global models, having a latitudinal and longitudinal grid size of ~2 degrees, have not had sufficient spatial resolution for urban scale studies; therefore, they were applied only for global-scale assessment instead of regional and/or urban scale (see Reference [7]).

### 2.1. Dataset and WBGT Calculation

Over the past decades, with the advancement in satellite remote sensing and climate model, climate reanalysis data have been widely used in many metrological fields, such as temperature and precipitation for forecasting. Reanalysis products used the fundamental physics law to combine model with observations around the world into a completed gridded global dataset which can effectively compensate for the lack of direct ground-based measurement.

This study used environmental data obtained from ERA5, the latest gridded reanalysis product produced by the European Centre for Medium-Range Weather Forecasts, as a representation of satellite-observational data on the global scale. Although several global atmospheric reanalyses are available (e.g., MERRA-2, JRA-55, and CFSR), ERA5 have been shown to be the best or amongst the best performing reanalysis products [16,17]. The spatial resolution of each grid cell is 0.25 × 0.25 degrees latitude and longitude. Other variables used to calculate the hourly inferred WBGT (hereafter, WBGT* to differentiate it from the instrumentally measured WBGT) include the following: surface downward short-wave radiation (Wm^−2^), 2 m temperature and dewpoint temperature (K), and 10 m surface wind in both U and V components (ms^−1^).

We used the Liljegren approach [18] to calculate the outdoor WBGT*, including the effect of background metrological variables, such as wind speed and radiation, which are sensitive to climatic changes. The Liljegren equation has proved to be the most reliable method for calculating the outdoor WBGT [19]. Equation (1) shows the WBGT* equation.

WGBT* = 0.7 Tnwb + 0.2 Tg + 0.1 Ta(1)

where Tnwb is the natural wet bulb-temperature (°C), Tg stands for the globe temperature (°C), and Ta represents the ambient air temperature (°C). While Ta can be obtained as the 2 m temperature directly from the ERA5 output, Tnwb and Tg have to be calculated by using an iterative solution method from the dew point temperature, relative humidity, wind speed, and intercepted radiation [17]. The following ERA5 variables and their long names were used: mean surface downward short-wave radiation flux (msdwswrt, ms^−2^), 2 m near-surface temperature (t2m, K), 2 m dewpoint temperature (d2m, K), 10 m U wind component (uwd, ms^−1^), and 10 m V wind component (vwd, ms^−1^).

(2) Td=d2m−273.15

(3) Ta=t2m−273.15

(4) $$\mathrm{RH}=100\times e^{\left(17.27\times \left(\mathrm{Td}/\left(237.7+\mathrm{Td}\right)-\mathrm{Ta}/\left(237.3+\mathrm{Ta}\right)\right)\right)}$$

(5) $$ws=\sqrt{\left(uwd^{2}+vwd^{2}\right)}$$

mrd = msdwswrt(6)

The program used to calculate natural wet-bulb and globe temperature was available at https://github.com/mdljts/wbgt/blob/master/src/wbgt.c.original (accessed on 29 June 2022):

Tnwb(:,i,j) = (/fTwb(Ta, Td, RH, AtmPressure, ws, MinWindSpeed, rad, propDirect, ZenithAngle, 1)/)(7)

Tg(:,i,j) = (/fTg(Ta, RH, AtmPressure, ws, rad, propDirect, ZenithAngle, MinWindSpeed)/)(8)

where AtmPressure is atmospheric pressure (101 kPa), MinWindSpeed is minimum wind speed upsets log function (0.1 ms^−1^), and propDirect is proportion of direct vs. diffuse radiation (0.8).

The italic font marks variables we set at constants in calculation, and the zenith angle is defined as angle between the sun and the vertical that varies with time and latitude. The Zenith angle in this study was obtain using solar position calculator maintain by NOAA https://gml.noaa.gov/grad/solcalc/azel.html (accessed on 29 June 2022).

### 2.2. WBGT Flag Conditions

The WBGT flag system was adapted by the US military as a safety guideline to determine the duration which individuals could work safely in hot and humid outdoor conditions [20]. This flag system has also been used to establish work–rest cycle recommendations for different levels of workload (i.e., US CDC report (2016) [21]) to ensure the safety of the laborer. Following the WBGT definition, increases in heat stress will result in a reduction in working time in order to avoid heat-related working hazards. In this study, labor-rest hours were calculated by using the standard for heavy work (600 W), which can be viewed as an upper limit for reduction in labor capacity. Table 1 shows the WBGT heat categories and corresponding hourly work–rest times. The work–rest cycle is meant to protect healthy adults by allowing them to carry a safe workload under environmental heat stress; it also allows us to estimate the potential economic impact of heat stress in terms of labor-hour reduction.

### 2.3. Urban Areas

Given the large proportion of the world population that lives in urban settings, the goal for this study was to examine variations in heat stress in heavily populated metropolitan areas during the past decade. We chose metropolitan areas mainly based on population counts in different regions. Metropolitan areas in Asia and North America (Europe) are defined as a 200 (100) km by 200 (100) km area that translates into 8 × 8 (4 × 4) grid cells. The area extents were estimated from the averaged city size in Google Earth images. All ERA5 metrological parameters used in the WBGT* calculation represent the areal average from the land-covered grid cells. The cities selected for analysis have to satisfy the following criteria: (1) grid cells have to be at least 70% land covered and on gentle terrain to avoid data assimilation bias, and (2) daily maximum and minimum temperatures have to evenly distributed in these grid domains. The selected cities are listed in Table 2, and examples of selected metropolitan areas are shown in Appendix A. This study used ERA5 data instead of ERA5-land with higher resolution (0.1 × 0.1 degree) data to save on computational costs. The WBGT* of the selected cities calculated from the two different datasets produced identical trends, with the differences in values all being within 5%. It should be noted that using the areal average as applied in this study could possibly lead to an underestimation of the WBGT* with respect to the conventional instrumental-measurement-defined WBGT due to the averaging procedure. Thus, in our view, the WBGT* represents a general condition for a metropolitan area rather than for a particular spot. Although urban land use is considered in ERA5 data, detailed settings such as for the building canopy and greenspace planning were not included [17].

## 3. Results

### 3.1. Continental Scale WBGT*

**Figure 1:**
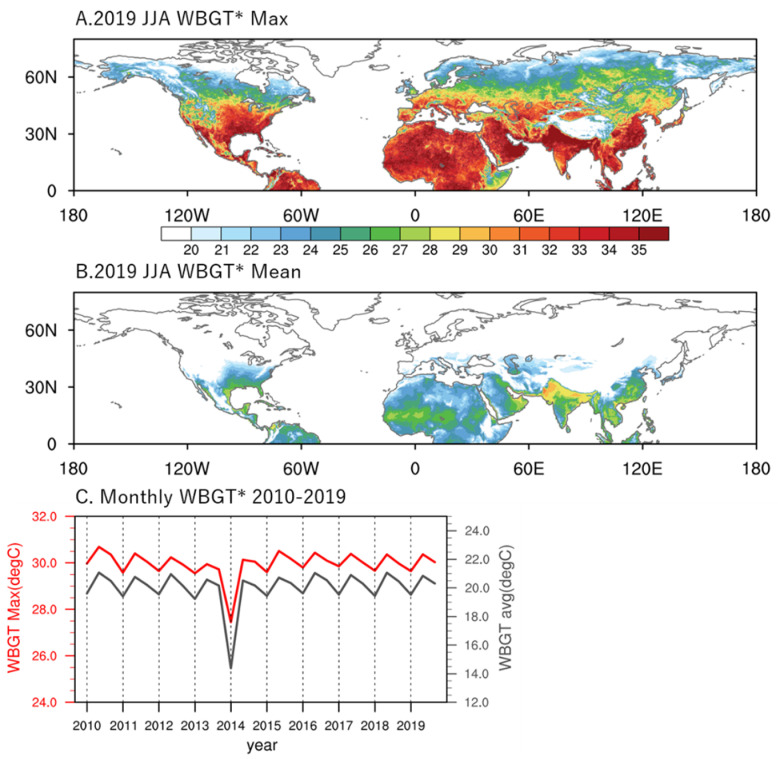
(**A**) Summer (June–August) averaged maximum WBGT* (°C) for the northern hemisphere in 2019. (**B**) Same as in (**A**), except for mean WBGT*. (**C**) Northern hemisphere land-only summer monthly averaged mean and maximum WBGT* during 2010–2019. The mean and max values showing in the figure are 50 and 98 percentiles, respectively, of the WBGT* data range.

The WBGT* was calculated based on an hourly resolution, using ERA5 reanalysis for 2010–2019. Figure 1 shows the summer mean and maximum WBGT* for the northern hemisphere in 2019. The highest WBGT* can be observed in Africa, the Middle East, South and Southeastern Asia, and Eastern North America, as expected from the mean climatology data. A general boundary for 30 °C maximum WBGT* is located at around 50° N, but the maximum WBGT* can exceed 35 °C in many regions (Figure 1A). In contrast, the mean WBGT* barely exceeds 26 °C, with high-value areas located in tropical and subtropical areas (Figure 1B). The large difference between the mean and maximum WBGT* is indicative of a strong influence of the diurnal cycle and highlights the importance of impact studies to consider daily extremes on a diurnal timescale.

**Figure 2:**
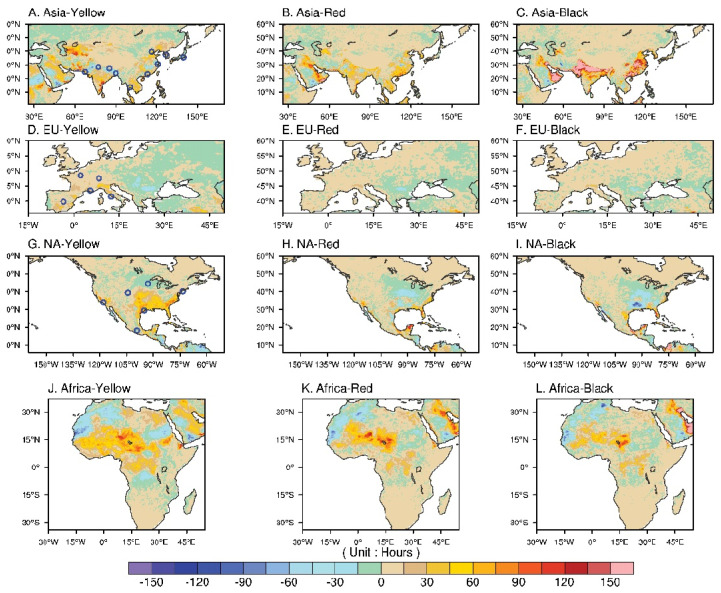
Difference in JJA rest hours between the periods of 2015–2019 and 2010–2014 (former minus the latter): (**A**–**C**) difference in rest hours due to yellow, red, and black flag conditions in continental Asia; (**D**–**F**) same as in (**A**–**C**) but for Europe; (**G**–**I**) same as in (**A**–**C**) but for North America; and (**J**–**L**) same as in (**A**–**C**) but for Africa. Blue dots on (**A**,**D**,**G**) show the geographic locations of selected urban cities in this study.

On the hemispheric scale, Figure 1C demonstrates the mean and maximum June–August (JJA) WBGT* over land each year. The most striking feature appears in 2014, which was an exceptionally cold year, as is evident in both the observations [22] and model simulations [23], especially in North America, and was often referred as the 2014 North America cold wave caused by the southward movement of the Arctic polar vortex. Other than the negative exception in 2014, the summer mean (max) WBGT* falls ~19–21 (29–31) °C, with July being the warmest month. We kept 2014 in the calculation because the summer 2014 still had a substantial number of days, particularly in South Asia, that had WBGT* flag days/hours. On the seasonal-to-monthly timescale, this corresponds to no flag or to a green flag for the maximum WBGT*. There is no apparent trend in hemispheric mean and maximum WBGT* during 2010–2019. As the WBGT is closely related to occupational heat hazard, we intend to highlight the potential economic impact on the urban scale by converting the WBGT* values to the number of rest hours needed based on heat category to assess potential labor hour reduction due to increasing heat stress. Figure 2 shows the difference in JJA rest hours (local time 08:00–18:00) between the periods of 2015–2019 and 2010–2014 (former minus the latter). The rest minutes for different flag categories derived from hourly data throughout the entire summer were added together and converted into hours. Climatological heat stress modeling/observations suggest relatively hot conditions in South and Southeastern Asia, the Eastern Arabian Peninsula, and Western Africa.

A comparison of the period from 2010–2014 to 2015–2019 showed that the continental Asia experienced some reduction in low-level yellow-flag warnings (Figure 2A), a slight increase in medium-level red-flag warnings (Figure 2B), and a dramatic increase in high-level black-flag warnings (Figure 2C) in the latter half of the decade. This might be indicative of a shift in the warming pattern from lower- to higher-level flag warnings and can most readily be seen in the Indo-Gangetic Plain and Eastern China (Figure 2C). Rest hours (labor reduction) associated with this pattern could exceed 150 h, equivalent to ~4 weeks of standard 8 h working days. A possible mechanism for this phenomenon is presented in the discussion in Section 4. In Europe, minor changes in WBGT* flags were observed, except for in the Alps region (Figure 2D–F). In Africa, WBGT* flag warnings showed a minor reduction in Northern Africa but an increase over the Sahel region and northern parts of the Sudanian savanna, resulting in a wide range of 30–100 resting hours (Figure 2J–L). In contrast to other major continental areas in the northern hemisphere, North America experienced some increase in yellow-flag warnings (Figure 2G) but a reduction in red- and black-flag times (Figure 2H,I). The Midwest experienced less heat stress, while the southeast and Gulf Coast experienced some low-level heat stress (Figure 2G). The pattern described seems to have a boundary at ~40° N. Little change was found in the Western Pacific coastal regions and Central Rockies.

Generally, South and Southeastern Asia were the global hotspots in the latter half of the 2010s, according to the aforementioned WGBT* flag pattern. This region has the potential for the most significant heat-stress-related economic impact and, more important, is home to many of the largest cities in the world based on population size.

### 3.2. Urban Scale WBGT*

To quantify the implications for potential urban heat hazard, we zoom in on the WBGT* on an urban scale by averaging meteorological parameters in a defined city domain (see Section 2.3 for definition) for the urban mean WBGT*. With human exposure in mind, urban areas in different continents were selected mainly based on total population count. Table 2 lists the population and area size of the chosen cities. Details of the criteria and data quality control are described in Appendix A. We applied the concepts of the heat category and work–rest cycle to assess the impact of outdoor activity and occupational labor safety. This work–rest cycle is designed to reduce the risk of overheating or hyperthermia and its associated ill effects. We calculated the hourly WBGT* and resulting rest minutes based on the standards for heavy work. Note that heavy work (600 W) is equivalent to the occupational lifting and carrying which are typical for outdoor laborers and typical vigorous outdoor exercise such as running output is about 4.0 Wattskg^−1^. This standard is meant to protect healthy adults, to determine a safe workload under environmental heat stress, and to also allow us to obtain a quantitative estimate of the reduction in labor capacity.

#### 3.2.1. Asia

**Figure 3:**
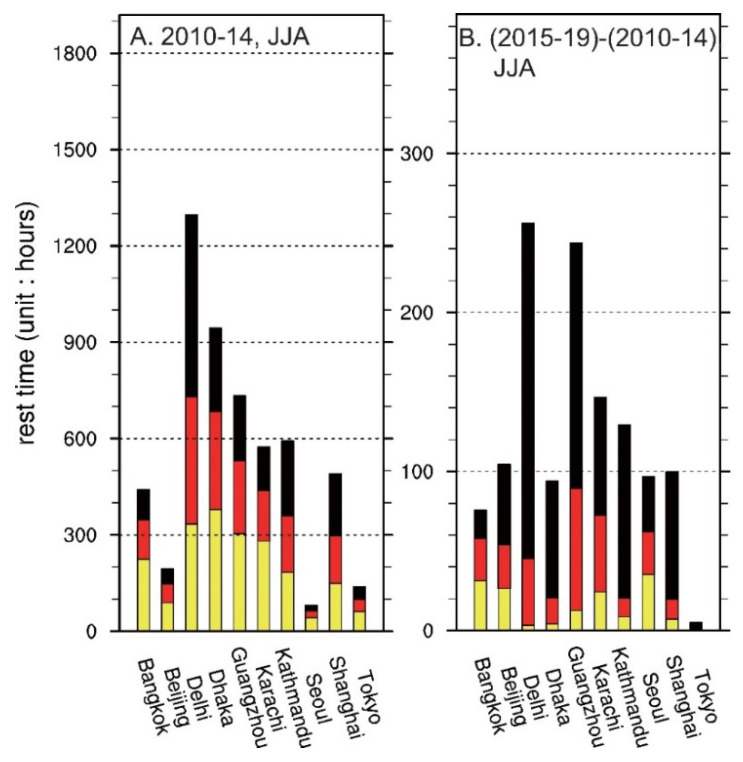
(**A**) Accumulated JJA rest hours during 2010–2014 for the selected Asian cities. Yellow, red, and black colors correspond to rest time due to the particular flag condition; (**B**) same as in (**A**) but showing the difference between the periods of 2015–2019 and 2010–2014 (former minus the latter).

Figure 2 (blue circles) and Table 2 show the geographic locations and urban population, of the selected cities. It can be seen that, of the Asian cities, Seoul and Tokyo have the lowest number of rest hours during the summer months in 2010–2014, i.e., ~100 h, which is equivalent to ~20 h a year or ~13 min per day (Figure 3A). The Chinese cities of Beijing, Shanghai, and Guangzhou show an accumulated rest time ranging from ~200 to ~750 h, with the differences generally reflective of geographic latitude. The striking feature is that cities in South Asia have the highest heat stress among all selected cities worldwide. New Delhi, located in a continental setting, accumulated ~1300 resting hours (170 min/day) per heat-stress-flag guideline. Karachi, Dhaka, and Bangkok, in coastal or maritime environments, also showed an accumulation of about 600, 950, and 450 resting hours, respectively, during the summer months from 2010 to 2014. Kathmandu, standing at an elevation of ~1400 m in the Himalayan foothills, also showed a high number of accumulated rest hours due to strong clear-sky solar radiation.

To assess the inter-annual variability of potential heat stress, we compared the difference in resting hours for the next 5-year period, from 2015 to 2019. It was expected that this period would be warmer, with longer resting times, especially since four out of these five years were among the top five hottest years on record globally, prior to 2020 (three out of five years were among the ten hottest years on record for the period from 2010 to 2014). The cities Beijing (100 h), Kathmandu (140 h), Karachi (150 h), Gaungzhou (240 h), and New Delhi (260 h) showed increases in resting hours of >110 h. We should also note that most of the increases were due to a sharp climb in the black-flag category. For example, more than 80% of the increase in rest hours in New Delhi and Kathamndu was from black-flag warnings. We discuss a possible mechanism in Section 4.

#### 3.2.2. Europe, Africa, and North America

**Figure 4:**
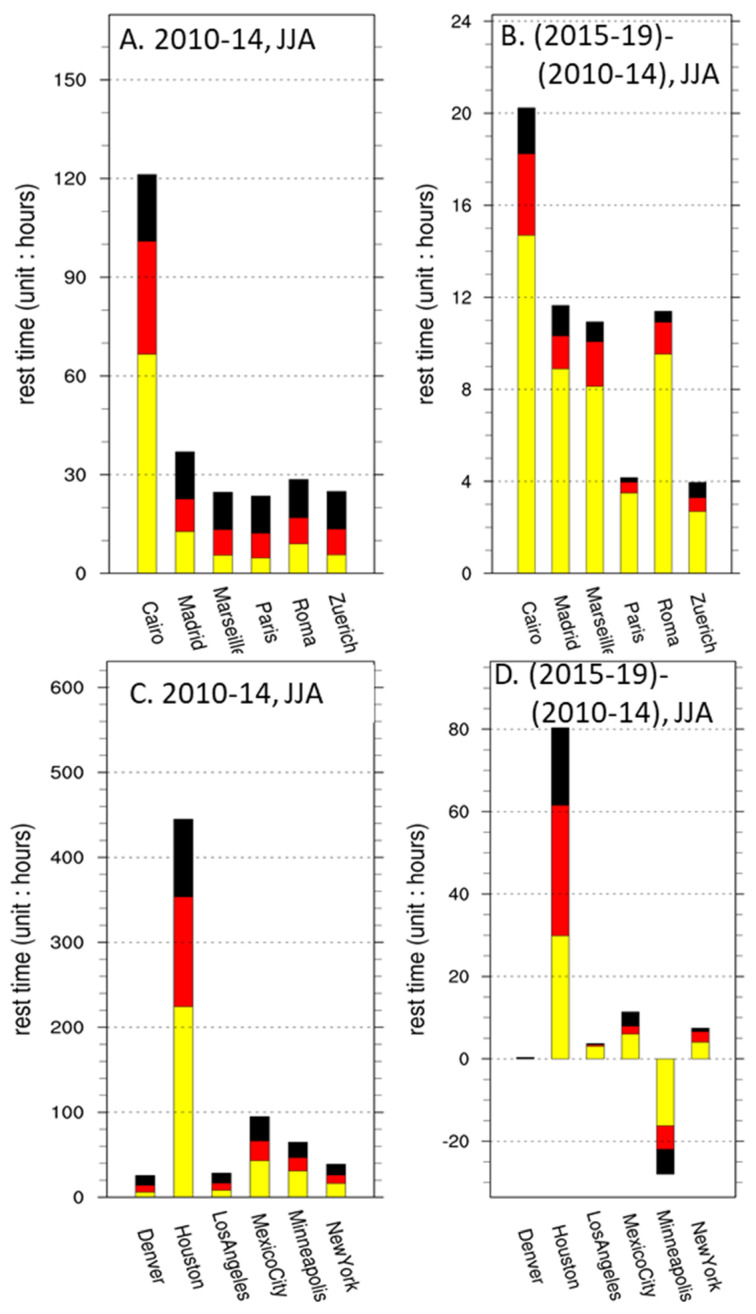
Same as in Figure 3, except (**A**,**B**) for European cities and (**C**,**D**) for North American cities. Note the differences in scale for the rest-hour difference.

Although ERA5 has captured some summer heat waves, such as the one that occurred in 2019 in Europe, the WBGT* summer resting times in the selected European cities within this decade were all within fifty hours (Figure 4A). The number of resting hours was slightly higher in Southern Europe, and there were also increases during 2015–2019, as expected from mean warmer climatic conditions and heat-wave data. The increase in rest hours in European cities was mostly from lighter-degree yellow-flag warnings. The African city Cairo is apparently having higher rest time than the European cities because of its geographic location in the subtropical climate zone (Figure 4A,B). The partition of rest time due to different flags is similar to Southern European cities, and the increase in rest hours is mostly due to yellow flags, thus suggesting regional consistency.

Similar to Europe, the North American WBGT*-categorized urban resting hours were also less than 100 h, except for Houston on the Gulf Coast in the period from 2010 to 2014. Houston experienced an 80 h increase of rest time, more or less equally distributed among flags. It is interesting to note that Minneapolis in the Midwest showed a decrease in rest hours. This is a regional-scale feature (Figure 2G–I) that is also observed in other Midwestern cities such as Chicago.

Due to latitudinal location, Europe and North America continents are located in the temperate climate zone, thus making the threat of heat stress much lower in European and North American urban areas compared to that in tropical Asian cities. This result is consistent with continental-scale observations described in Section 3.1 and implies that the heat hazard in these areas might be the result of short-term events, such as heat waves varying on a daily time scale, instead of an accumulated rise in seasonal temperature.

## 4. Discussion

### 4.1. South Asian WBGT*

Although observational and reanalysis data are all consistent with recent global warming trends, the pattern and amplitude of near-ground temperature changes remain complex. It has been difficult to quantify what temperature changes should be considered as substantial in a particular city relative to elsewhere in the world. For this reason, it has also been difficult to identify those regions or urban areas that are most vulnerable to heat stress. Since heat stress is closely tied to health, safety, economic factors, transportation, comfort levels, and many other concerns, any substantial change will have an impact on all aspects of human activity [24]. The improvement in the development of reanalysis products has finally reached the point where they allow sufficient spatial resolution for urban-scale research. This can also make up for the limitations of station coverage and, most important here, allow the inclusion of all regions under a standardized reference framework. 

In the previous section, we described conditions in South Asia, especially the Indo-Gangetic (IG) region, as this was a global hotspot during the second half of the 2010s. Here, we explore meteorological conditions in the IG region and discuss the probable reason why South Asia heat stress and WGBT* flag resting times increased drastically relative to the other cities presented in Section 3. Similar causal mechanisms generally also apply to other urban cities in South Asia. It is notable that heat stress in other regions (Europe, North America, Africa) is consistent with the superimposed global warming trend, and the magnitudes of WBGT* increases among cities were within 1σ of interannual variability within the entire ERA5 data range since 1979. We therefore attribute the enhancing urban heat stress as a result of the warming trend.

South Asia, including the IG river plain, not only stands out in our results, but is also important in the real world because it is home to a significant proportion of world’s population. Furthermore, the IG region contains ~40% of the total population of India and produces about half of its agricultural yield. Climatologically, the areal hydrological conditions are controlled by both the strength of the Indian summer monsoon and melting of the Himalayan snowpack. Recent anthropogenic climate influence studies raise concerns about aerosol effects on the local radiative energy balance arising from mineral scattering and absorbing black carbon from biomass burning [25,26,27]. These factors are well represented in the EAR5 data, which include forcings for sea surface temperature, sea ice, total solar irradiance, ozone, greenhouse gases, and aerosols. The CMIP5 simulations conducted by the World Climate Research Program (WCRP) [17] consider the aforementioned processes in assimilated temperature and other metrological parameters used to compute the WBGT*.

It can be seen in the ERA5 data that, during the 2010s, normalized rest hours in New Delhi, Kathmandu, and Dhaka were negatively (positively) correlated with cloud forcing (latent heat) (Appendix B). It is suggested that the increase in heat stress due directly to global warming was magnified by regional aerosol input that has an impact on cloud scattering/absorbing and the aerosol radiative effect, as well as changes in atmospheric circulation and associated rainfall. Considering the unique environmental characteristics of high aerosol counts and summer monsoon rainfall in South Asia, the correlations are consistent with a direct aerosol effect and precipitation related surface cooling. The correlation with cloud forcing decreased in cities (i.e., Karachi) away from the IG region, where seasonal monsoon circulation and diurnal land–sea breezes might be more important and the input of aerosols due to biomass burning is lower. It should be noted here that this study discusses decadal variations in WGBT* warming, which is different from the long-term cooling trend in the IG region described by Joshi et al. (2020) [28].

**Figure 5:**
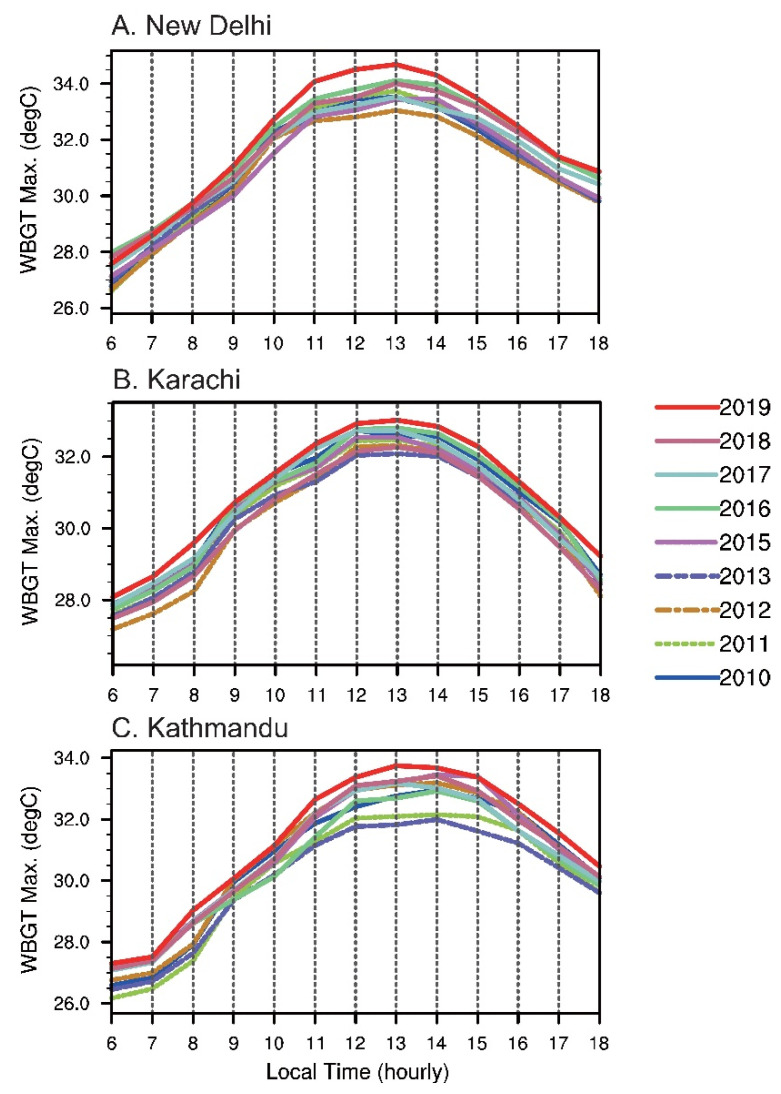
Decadal variability of diurnal cycle of JJA averaged-maximum WBGT* during the 2010s. The cold snap in the year 2014 described in Section 3.1 is not shown here for the WBGT* scale: (**A**) New Delhi, (**B**) Karachi, and (**C**) Kathmandu.

As a consequence of extensive warming, South Asia has experienced an increase of up to ~20% in WBGT* flag resting hours, mostly due to black-flag times. The phenomenal increase in resting hours during 2015–2019 is likely a result of longer high-risk exposure times during the daytime. Figure 5 shows the diurnal cycle for the JJA averaged-maximum WBGT* during the 2010s for New Delhi, Kathmandu, and Karachi. It can be seen that the second half of the decade, from 2015 to 2019, is generally hotter than the first half, and 2019 is the hottest year based on evidence from observational records. The maximum WBGT* in New Delhi reached the black-flag threshold (32.2 °C) about one hour earlier in the morning and 1.5 h later in the evening between the warmest and coolest year of the 2010s. Similar diurnal changes can be found in Karachi, but with milder WBGT*, resulting in a more even distribution of flag colors (Figure 3). In Kathmandu, there were many more hours with a WBGT* higher than 32.2 °C after 2015, a phenomenon rarely seen from 2010 to 2014.

### 4.2. Heat Hazard

The WBGT* and associated resting hours in selected cities clearly show the high level of the heterogeneity of the degree of heat stress in different geographic locations. Although this study shows decadal variation rather than long-term trends and/or projections, our results point out the existence of a high-level threat. Furthermore, based on an Intergovernmental Panel on Climate Change assessment report [2], continued emissions of greenhouse gases, regardless of the scenario, will result in further warming. There is a high level of confidence that there will be more frequent extreme hot days and heat waves in the near future. Reanalysis-product results, together with model projection results, could be useful in urban planning and project designs to consider potential future heat hazards. 

Heat stress and adverse health-related consequences have been well documented in the literature (see Spector et al. [29] and the references therein). Outdoor occupational injury is one of major concerns in these studies. Fatima et al. [30] shows that occupational injury increases by 1% per degree Celsius increase in temperature over the threshold and 17.4% during heat waves. The authors further identified outdoor workers being at particularly high risk. Overheating conditions are a potential labor safety problem which also has economic implications. Using work-related-injury-insurance-claim data, Ma et al. [14] showed an increase in injury claims of 4.8% with rising WBGT that resulted in a 4.1% increase in insurance payouts. They further pointed out that workers in small businesses and with lower educational attainment were particularly sensitive to environmental heat stress. Figure 2 shows the increase in heat stress to be most significant in South and Southeastern Asia, where many workers employed in outdoor environments have limited legal protections and access to healthcare. Together with the aforementioned studies and our results, the implication is that the effect of heat exposure in these regions therefore represents both a societal economical problem and a related inequality issue. The provision of legal protections for the work/rest cycle might be a necessary approach to prevent economic costs and social injustice.

In this study, the resting hours (Figure 3 and Figure 4) are calculated for local times of 08:00 to 18:00 in consideration of summer daylight working hours instead of a typical 8 h working day. The number of outdoor work hours might therefore be slightly overestimated when compared to the typical 09:00–17:00 h workday. However, extending the time an extra hour in the morning and in the evening should have the least impact on the total accumulation of resting hours, because these parts of the diurnal cycle are relatively cool. For the same reason, shifting the working hours earlier or later during a day may lead to a significant reduction in the estimated heat hazard. Our result suggests that shifting the beginning and end of working hours within the day might be one way for occupational safety management decision-makers to reduce the workers’ heat hazard, even in the hottest areas in South and Southeastern Asia. Similar practices were adopted during the 2021 Tokyo Olympics, as intensive heat and humidity forced the organizer to move competitions to the late afternoon.

The boreal summer WBGT* data presented here are consistent with respect to conventional climate zones. These data imply a need to establish guidelines for local heat-acclimation conditions. The WBGT flag represents a first-order human-body response to heat stress without acclimation considerations. A black flag in Europe might be a rare and high-stress occurrence but simply be part of the diurnal and seasonal routine in India. Moreover, healthy heat-acclimated human bodies can tolerate extended heat stress, given sufficient water supply and protection from the sun [31,32]. Statistical results also show an increase in the minimum mortality temperature in the period from 1995 to 2017 in the Netherlands [33], indicative of long-term adaptation to heat stress. As each climate zone has its unique metrological features of temperature, humidity, and radiation, strategic adaption is therefore not universal but locally dependent.

## 5. Conclusions

This study used the WBGT* derived from ERA5 data to replace conventional in situ measurements in order to represent environmental heat stress from the continental to the urban scale during the decade from 2010 to 2019. The results indicated that South and Southeastern Asia were exposed to severe heat stress and environmental hazards for outdoor safety, according to the WGBT flag warnings, which can be explained by changes in local precipitation and cloud coverage. Europe and North America experienced relatively mild heat stress. On the urban scale, the loss of labor hours produces a nontrivial economic cost, which also has implications for warming adaptation and social inequality.

## Figures and Tables

**Table 1:** WBGT heat category and corresponding rest time per hour.

Heat/Flag Category	WBGT Index (°C)	Resting Time (min h^−1^)
No flag	<29.4	N/A
Yellow	29.4–31.1	15
Red	31.1–32.2	30
Black	>32.2	45

**Table 2:** List of metro city populations in this study. Data are based on Demographia World Urban Area (2019).

		Population (Thousand People)
Asia	Tokyo/Japan	37,977
	Delhi/India	29,617
	Seoul/South Korea	21,794
	Bangkok/Thailand	17,066
	Dhaka/Bangladesh	15,443
	Karachi/Pakistan	15,400
	Kathmandu/Nepal	3045
	Shanghai/China	22,120
	Guangzhou/China	20,902
	Beijing/China	19,433
Europe	Paris/France	11,020
	Madrid/Spain	6026
	Roma/Italy	3995
	Marseille/France	1605
	Zurich/Switzerland	805
Africa	Cairo/Egypt	10,025
North America	Mexico City/Mexico	20,996
	New York/USA	20,870
	Los Angeles/USA	15,402
	Houston/USA	6406
	Minneapolis/USA	2855
	Denver, CO/USA	2690

**Figure A1:**
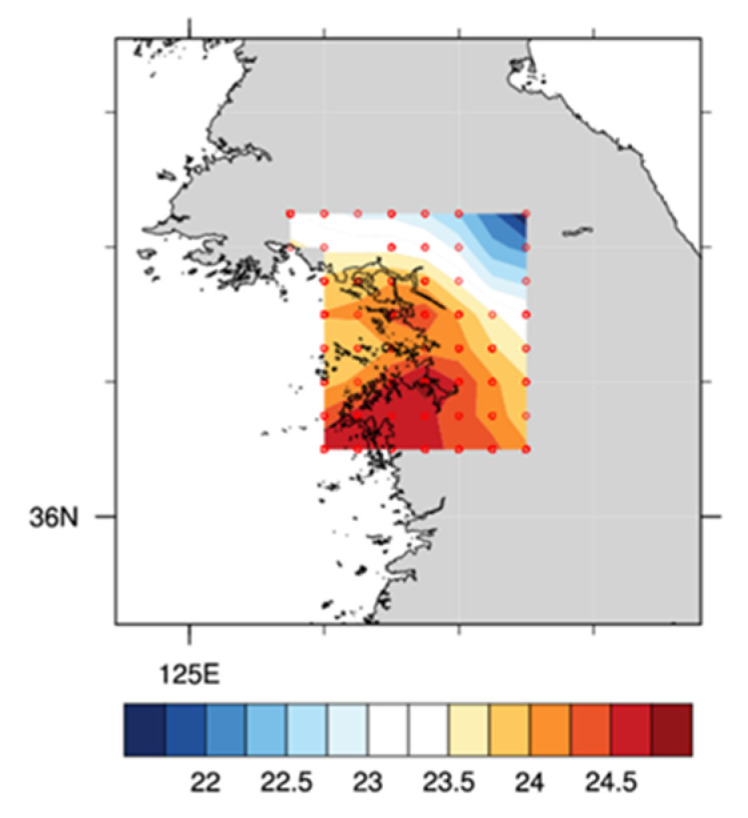
WBGT* in Seoul, South Korea at 12:00 local time on 1 July 2017.

**Figure A2:**
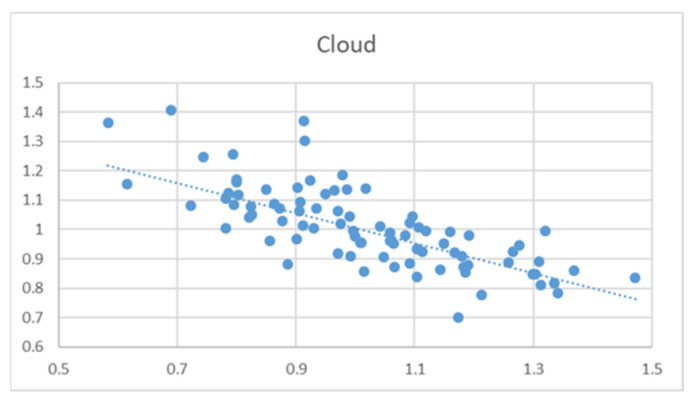
Normalized monthly mean cloud forcing (*y*-axis) vs. rest hour (*x*-axis) during 2010–2019 for New Delhi, Kathmandu, and Dhaka.

**Figure A3:**
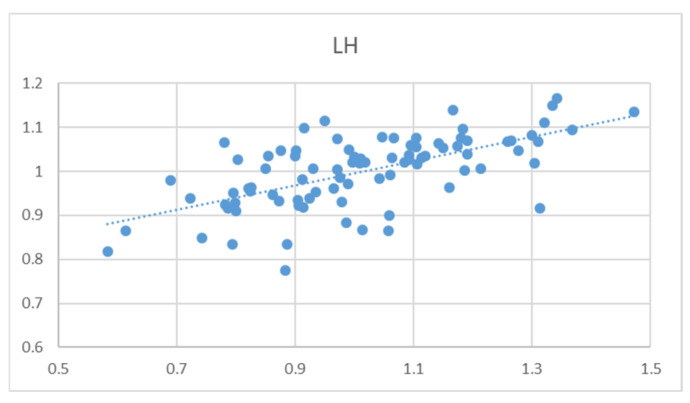
Normalized monthly mean latent heat flux (*y*-axis) vs. rest hour (*x*-axis) during 2010–2019 for New Delhi, Kathmandu, and Dhaka.

## Data Availability

Not applicable.

## Appendix A. WBGT* in Selected Urban Regions

We define a metropolitan area as a 200 km by 200 km area that translates into 8 × 8 grid cells. Cities with too many “water body” grid cells (e.g. Chicago surrounded Lake Michigan) were omitted from our selection to avoid bias from ERA5 model land-sea orography mapping. After removing the land-sea mapping, daily maximum and min temperature should randomly distribute in the defined domain to minimize artificial dominance made by particular surface type.

Here, we use Seoul, South Korea as an example. The following Figure A1 shows WBGT* at 12:00 local time on 1 July 2017. Only land grids were considered in the calculation. Red dots denoted grid cells that has “recorded” highest WBGT* in the defined domain during June-July-August in 2017. City was omitted if red dots were skewed to any particular region in the defined domain.

## Appendix B. Cloud Forcing and Latent Heat Correlation with Rest Hour

We calculate cloud forcing by subtracting mean surface downward short-wave radiation flux (msdwswrf) from mean surface downward short-wave radiation flux clear sky (msdwswrfcs) to represent direct aerosol absorbing and scattering in the atmosphere column. This assumption assume aerosol particle is the major source of shortwave radiation damping in the top-down in the atmosphere. Latent heat flux was directly taken from ERA5 output parameter, mean surface latent heat flux (mslhf).

To comparing urban cities in different metrological setting, cloud forcing, latent heat flux and resting hours were normalized against cities own mean during 2010–2019. We obtain monthly mean for each city during the 2010s as such each city would have 30 data point in this correlation. Figure A2 shows scatter plot of normalized cloud forcing (*y*-axis) against rest hours (*x*-axis) for New Delhi, Kathmandu, and Dhaka in IG plain. The negative relationship shown here suggest grater cloud forcing presumably caused by high aerosol loading in the atmosphere would led to more scattering of shortwave radiation and cooling in local surface temperature that can be reflected in local resting hours.

Another factor that has great influence on surface temperature is the balance between precipitation and evaporation that can be represented by the amount of net latent heat at the land surface and atmosphere interface. Greater latent heat flux here means drier condition. The IG region receives seasonal rainfall from Indian summer monsoon that brings in moisture landward from Indian ocean. As precipitation effectively cools the surface temperature, the positive correlation between latent heat (*y*-axis) and rest hours (*x*-axis) suggest an intuitive relationship of dry and hot seasonal condition in the IG region (Figure A3).
